# Exosomes: Mechanisms of Uptake

**DOI:** 10.5772/61186

**Published:** 2015-07-17

**Authors:** Kelly J. McKelvey, Katie L. Powell, Anthony W. Ashton, Jonathan M. Morris, Sharon A. McCracken

**Affiliations:** 1 Division of Perinatal Medicine, Kolling Institute of Medical Research, University of Sydney at Royal North Shore Hospital, St Leonards, NSW, Australia; 2 Pathology North, NSW Health Pathology at Royal North Shore Hospital, St Leonards, NSW, Australia; 3 Department of Obstetrics and Gynaecology, Royal North Shore Hospital, St Leonards, NSW, Australia

**Keywords:** Exosome, immunology, endocytosis

## Abstract

Exosomes are 30–100 nm microvesicles which contain complex cellular signals of RNA, protein and lipids. Because of this, exosomes are implicated as having limitless therapeutic potential for the treatment of cancer, pregnancy complications, infections, and autoimmune diseases. To date we know a considerable amount about exosome biogenesis and secretion, but there is a paucity of data regarding the uptake of exosomes by immune and non-immune cell types (e.g., cancer cells) and the internal signalling pathways by which these exosomes elicit a cellular response. Answering these questions is of paramount importance.

## 1. Introduction

In the 1960s, Robert Feynman implored researchers to try to develop nanotechnology with the ability to interact with the human body at the cellular level [1]. Since the 1990s, nanomedicines generated from polymeric or liposomal nanoparticles—encapsulating or adsorbing one or more drugs—have been assessed in clinical trials and implemented in the clinic, for the treatment of cancer, HIV/AIDS, malaria, and tuberculosis. Biologically, exosomes offer a viable “natural” therapeutic avenue for immune modulation. Exosomes are nanoscale (30–100 nm) vesicles containing lipid, protein and RNA species in a single biological unit. They are present in the intracellular space and in body fluids (including plasma, saliva, urine, pleural ascites, amniotic fluid, cerebrospinal fluid, colostrum, breast milk and semen), and may act locally or from a distance, through the secretion of soluble factors or cell-cell contact.

Previous reviews have detailed exosome biogenesis, composition, and the cellular response of T cells to tumour-[2], dendritic cell (DC)- [3–5], and placenta-derived exosomes [6]. This review highlights recent advances in these research areas of exosome function, but focuses on what is currently known about exosome targeting, internalization and elimination, with specific reference to T cells.

## 2. Exosome Biogenesis and Secretion

Exosome biogenesis starts with the invagination of the late endosomal limiting membrane into the lumen to form multi-vesicular bodies (MVBs). Consistent with the formation of other cell-derived vesicles, the formation of exosomes requires the evolutionarily conserved soluble *N*‐ethylmaleimide-sensitive factor attachment protein receptor (SNARE), Rab, coat complex subunit and Sec 1 proteins [7]. At this stage, MVBs contain “recycled” cell-surface proteins, and RNA and proteins derived from the cytoplasm. “De novo” proteins from the endoplasmic reticulum and Golgi complex may be directly sorted into the MVBs, guided by either (i) sequential action of the endosomal-sorting complex required for transport (ESCRT) machinery [8] (specifically AIP1/Alix/Vps31 and Tsg101/Vps23 [9]), or (ii) by a ceramide/tetraspanin-dependent pathway [10].

Most cells, including those grown in culture, are constantly releasing exosomes. In immune cells, this response can be enhanced by potent activation signals, such as antigenic, cytokine or mitogen stimulation [11]. Upon stimulation of the cell, MVBs polarize to the immunological synapse within 10 min of engagement [12], and are released into the extracellular environment by fusion with the plasma membrane. The process of MVB docking at the plasma membrane and subsequent fusion is regulated by Rab, SNAP and SNARE proteins. The Rab27b and Rab27 effector molecules, synaptotagmin-like 4 and exophilin 5, regulate docking of the MVBs at the plasma membrane [13]. Meanwhile, Rab11 and Rab31 [13, 14], the R-SNARE protein YKT6 [15], and the v-SNARE protein VAMP7/TI-VAMP [16] are implicated in the fusion event between MVBs and the plasma membrane. Rab27a has been shown to play a role in both docking and fusion [13, 14].

Notably, it is plausible that some exosomes are released by cells via direct outward budding and fission of the plasma membrane, analogous to shedding microvesicles and apoptotic blebs.

## 3. Exosome Cellular Recognition

While there is a large body of evidence relating to exosome biogenesis, our understanding of exosome internalization is in its infancy. Newer techniques such as fluorescent (Dil)-labelling and the PolyParticleTracker programme are being used to reveal the dynamics of exosomes during cellular internalization [17]. Below is a discussion of the current data on exosome internalization.

### 3.1 Free Floating

Exosomes released from cells circulate in body fluids at least for a short period of time. Intravenous administration of fluorescently labelled B cell-derived exosomes, via the lateral tail vein of mice, revealed that the half-life of exosomes in plasma was ~2 min [18]. However, exosomes were detectable in the spleen up to 2 hrs later. Following intranasal administration, exosomes have been found within the brain and intestine after 3 hrs [19]. In both studies, exosomes were found to co-localize with macrophages, and did so within 15 min of administration [19]. The data suggest that exosomes are rapidly sequestered by circulating monocyte/macrophages in the liver and spleen, though whether this is for clearance or cellular signalling is unclear.

From intravital video microscopy of capillaries in the ears of mice, in combination with computer modelling, it has been estimated that nanoparticles of the exosome size range (30–100 nm) randomly distribute within the blood vessel, drifting laterally from the red blood cell core toward the vessel wall where leukocytes localize [20]. This may partially explain the systemic diffusion of exosomes to sites distant from the secreting cells, and how exosomes may come into contact with monocytes/macrophages, as well as T cells. Similarly to liposomes, this could be mediated by the opsonization of exosomes during circulation [21]. Phosphatidylcholine—hydrolysed to lysophatidylcholine by calcium-independent phospholipase A2 (iPLA2) on the surface of exosomes from rat reticulocytes—binds natural IgM antibodies and complement component C3, promoting phagocytosis [22]. Other opsonins present on the surface of exosomes, including phosphatidylserine and lactoadherin (MFG-E8) [23], may explain the co-localization of exosomes with macrophages noted in abovementioned studies [18, 19]. While T cells are not considered “professional” phagocytes, γδ T cells can phagocytose bacteria and synthetic beads via antibody opsonization and FcγRIII receptors [24].

An alternative explanation could be that exosomes use chemokines to attract leukocytes to their location. Exosomes express an impressive array of chemokines, which may attract T cells and other cell types [25]. These include CCL2, CCL3, CCL4, CCL5, CCL7, CCL20, CCL28, CXCL1, CXCL2, and CXCL16. Of these, CCL2, CCL5, CCL20, and CXCL16 are potent T cell chemoattractants.

### 3.2 Adhesion

A fundamental step for exosome-T cell communication is adhesion. Lymphocyte adhesion by exosomes requires the conformational change of integrins from a low to a high affinity status. This enables oligomerization of integrins and coupling with cytoskeletal elements, to facilitate the high avidity binding of the lymphocyte to the integrin-bound exosome [26]. The presence of ICAM-1 (CD54) on mature DC-derived exosomes is critical for efficient naive T cell priming, mediated by the leukocyte integrin LFA-1 (CD11a/CD18 or αLβ2) [27]. Other integrins that have demonstrated roles in exosomes-leukocyte adhesion are integrins β1 (CD29), α3 (CD49c) and αv (CD51), and lactadherin and vitronectin, which are ligands for αvβ3/β5 integrins [9, 28], [29]. While the initial binding/docking of T cells to exosomes is regulated by ICAM-1/LFA-1 [30], firm adhesion is further facilitated via αL (CD11a), α4 (CD49d), CD44 and ICAM-1 expression on leukocytes, and the expression of tetraspanins CD9 and CD81 on exosomes [29, 31].

Tetraspanins are highly conserved through evolution and have a role in adhesion, motility, signal transduction and cell activation. A number of tetraspanins have been reported on the surface of exosomes, including CD9, CD53, CD63, CD81 and CD82, although the combination and proportion of exosomes expressing a particular tetraspanin differs depending on the cell of origin. One example is the abovementioned observation that only ~50% of exosomes from activated platelets express CD63 [32]. Tetraspanins form heterobimolecular complexes with integrins (e.g., α3β1) [33], Ig superfamily members (e.g., ICAM-1, MICA, MICB) and co-receptor molecules (e.g., CD4, CD8, CD19, CD21) [26, 34], [35]. It is thought that the expression of tetraspanins on exosomes contributes to the spatial assembly for antigen recognition and may partially dictate the signal induced by the exosome. For example, TCR co-stimulation via CD9 resulted in only partial T cell activation before the T cell underwent apoptosis, while traditional co-stimulation via CD28 leads to the proliferation of T cells [36].

Other adhesion proteins are demonstrated to play a role in the capture of exosomes, including CD169 (sialoadhesin) on macrophages [18], and heparin sulfate proteoglycans on both U-87 MG glioblastoma cells [37] and 293T human embryonic kidney cells [38], while the latter may also bind cytotoxic and/or helper T cells.

### 3.3 Antigen Recognition

To date, molecular profiling and proteomic analysis has demonstrated that target cell specificity for exosomes appears to be dictated solely by a combination of antigen and MHC class I (Tc cells) and II molecules (CD4^+^ T cells) [39]. The expression of MHC molecules on exosomes is dependent on the expression of the molecule on the parent cell. While the microRNAs contained in some exosomes differ greatly to those of the parent cell [12, 40], the expression of MHC class I and II molecules is similar to that of the originating cell [41].

The expression of MHC class I molecules on exosomes induces a negative signal via inhibitory receptors on the recipient cell, such as ILT2, ILT4 and KIR2DL4, which promote the inhibition of CD8^+^ Tc cells and NK cell responses. MHC class I molecules are often found on exosomes in conjunction with other immune modulating molecules, such as the B7 molecules CD274 (B7H-1) and CD276 (B7-H3) [42], and the MHC class I-related molecules MICA and MICB, which down-regulate the NK cell-activating molecule NKG2D on immune cells [43]. Exosomes expressing HLA-G1 are secreted by melanoma cells [44], and may play a role in immune evasion, which enables escape from the immune response.

MHC class II molecules evoke a stimulatory signal and promote the proliferation and differentiation of CD4^+^ T cells. Exosomes from antigen-presenting cells such as DCs and B lymphocytes express MHC class II. Exosomes bearing HLA-DR1–haemagglutinin (306–318) complexes weakly activate HA/DR1-specific T cells; however, the incubation of HLA-DR1+ exosomes with DCs resulted in highly efficient stimulation of antigen-specific T cells [45]. It remains to be seen whether there is another as-yet-unidentified mechanism of cell targeting by exosomes.

## 4. Exosome Internalization

**Figure 1.**
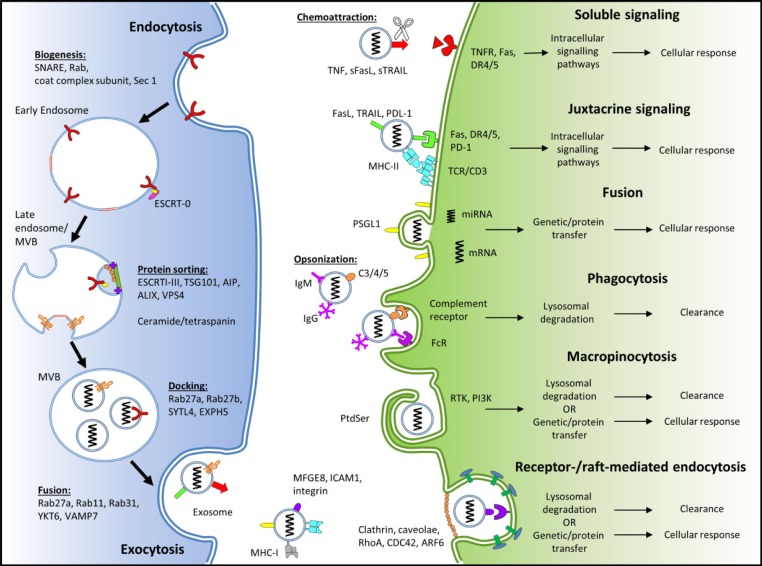
Schematic of exosome biogenesis, internalization and cellular response. The adhesion of exosomes to the recipient cell utilizes the interaction of various exosomal surface proteins and cellular receptors. Once bound, the exosome may (i) elicit transduction of the signal via intracellular signalling pathways and be released (*juxtacrine signalling*); (ii) fuse with the cellular membrane transferring protein and genetic contents, into the cytoplasm of the recipient cell (*fusion*); or (iii) be endocytosed via *phagocytosis, macropinocytosis* or *receptor-mediated endocytosis*. This figure was produced using Servier Medical Art, available from www.servier.com/Powerpoint-image-bank.

It is still unclear as to whether exosomes must be internalized by immune and non-immune cells in order to elicit cellular responses. For example, cellular responses elicited by RNA species rely on internalization. By comparison, cellular responses induced by membrane-bound or soluble FasL and TRAIL from exosomes do not require internalization, but are dependent on location and temporary adhesion for juxtacrine or soluble signalling. Equally, differing opinions as to the method by which exosome internalization occurs—either by fusion, receptor-mediated endocytosis, macropinocytosis or phagocytosis—exists within the literature. However, the latter two methods may represent mechanisms for the clearance of exosomes, rather than the elicitation of a cellular response. A summary of the internalization mechanisms for exosomes, and some of the key proteins involved, is provided in Figure 1.

### 4.1 Soluble and Juxtacrine Signalling

Soluble signalling involves the proteolytic cleavage of ligands from the exosomal surface or alternative splicing, while juxtacrine signalling requires the juxtaposition of ligands and receptors on the surfaces of the exosome and target cell. Membrane-bound FasL, TRAIL and TNF can be cleaved by metalloproteinases to form soluble cytokines. Nevertheless, it should be noted that the death ligands, soluble FasL and TRAIL, have a reduced pro-apoptotic activity when compared to that of the membrane-bound form [46, 47]. Exosomes from cultured placental explants or plasma from pregnant women have FasL and TRAIL on their membrane, and induce apoptosis in Jurkat T cells, via NF-κB, CD3ζ and JAK3 down-regulation [48][Bibr bibr49-61186]–[50] [Sharon McCracken, unpublished findings]. The same mechanism has been demonstrated for exosomes derived from tumours [51].

### 4.2 Fusion

Vesicle-cell fusion is the process by which a vesicle merges with the plasma membrane of a cell. Using a fluorescent lipid-mixing assay and membrane fusion assay, monocyte-derived microvesicles were demonstrated to bind and fuse with the plasma membrane of activated platelets, and to transfer proteins to the recipient cell, such as tissue factor and P-selectin glycoprotein ligand-1 (PSGL-1) [52]. In a similar experiment, exosomes from metastatic melanoma cells fused with the plasma membrane, which could have been inhibited by filipin [53]. Proteins were found to have a minor, possibly structural, role during fusion. Co-localization of exosomes with Rab53 or Lamp-1 suggested that exosomes are internalized and interact with cytoplasmic vesicles [53].

Unlike endocytosis, in which multiple mechanistic pathways have been detailed, the mechanism of cell-cell fusion is incompletely understood. The phenomenon may be regulated by tetraspanin complexes on target cells. Tetraspanins have a role in T cell activation and membrane fusion between sperm-oocyte [54, 55], myoblasts [56], mononuclear phagocytes [57] and mammalian viral-cell fusion [58].

While tetraspanin CD81, which co-localizes with CD4, has been shown to be involved in exosome release from HIV-infected T cells [59, 60], it has not yet been demonstrated whether tetraspanins are involved in exosome-T cell fusion. In the context of viral-cell fusion, tetraspanins inhibit cell fusion; CD9, CD63, CD81, CD82, CD151 and CD231 reduce HIV infection [61, 62] by inhibiting cell fusion [63, 64] and cell-cell transmission of viral particles [65]. The inhibition of cell-cell fusion of mononuclear phagocytes by CD9 and CD81 has also been reported [57]. Whether this is also true in the context of exosome-cell fusion remains to be seen.

It also seems plausible that, in a similar manner to leukocyte transendothelial migration, integrins are involved in exosome adhesion/attachment to the target cell, and following this, tetraspanin-enriched microdomains facilitate exosome fusion, which is alluded to in previous reports [66, 67].

### 4.3 Phagocytosis

Phagocytosis is an actin-mediated mechanism which requires the presence of specific opsonin receptors (i.e., FcR and complement receptors), scavenger receptors or toll-like receptors (Figure 1). While phagocytosis is typically performed by “professional phagocytes” such as macrophages and DCs, it can also be performed by “non-professional” cells, including γδ T cells [24]. Phagocytosis has been proposed as a means of exosome internalization. Not surprisingly, monocytic/macrophagic cell lines were able to internalize exosomes derived from erythroleukaemia (K562) and T cell leukaemia (MT4) cells more efficiently than “non-professional” phagocytic cells, including Jurkat T cells and 293T human embryonic kidney cells [68].

The phagocytosis of exosomes was shown to be dependent on the actin cytoskeleton, phosphatidylinositol 3-kinase (PI3K), and dynamin2 [68]. Notably, actin, PI3K and dynamin2 have all been implicated in both clathrin-mediated endocytosis [69], and phagocytosis [70]. Internalized exosomes co-localized with Lamp-1, lysobisphosphatidic acid and Rab7 [68] in late autophagosomes and/or endosomal and lysosomal vesicles [71]. Determining whether phagocytosis represents a true method of exosome internalization for the purpose of intercellular communication, or is merely a means of elimination, requires further research.

### 4.4 Macropinocytosis

During macropinocytosis, plasma membrane protrusions driven by actin filaments form an invagination which non-specifically endocytoses extracellular fluid and small particles. Reportedly, phosphatidylserine (PtdSer; Figure 1) on the surface of oligodendrocyte-derived exosomes activated macropinocytosis in a subset of microglia/macrophages without antigen-presenting capability [72]. Macropinocytosis of exosomes is dependent on Na^+^ and PI3K, with the inhibition of Na^+^−H^+^ ion exchange and PI3K activity by the pharmacological inhibitors EIPA and LY294002, respectively, reducing exosome uptake [73].

### 4.5 Receptor- and Raft-mediated Endocytosis

As the names suggest, receptor-mediated endocytosis and raft-mediated endocytosis require either a ligand on the exosomal surface to engage specific receptors on the cellular plasma membrane, or the presence of cholesterol- and sphingolipid-rich microdomains in the plasma membrane, respectively. The former, also called clathrin-mediated endocytosis, utilizes clathrin and adaptor protein 2 complexes which coat the membrane and induce the invagination of the membrane into a vesicle. The latter includes caveolae-mediated endocytosis, as well as the clathrin- and caveolae-independent endocytosis mechanisms RhoA-, CDC42-, and ARF6- regulated endocytosis, which utilize distinct combinations of dynamin, flotillin and/or Rab proteins [74]. The endocytosis of particles from the external environment or plasma membrane may be sent to lysosomes for degradation, or recycled back to the plasma membrane.

Exosomes released from cultured rat adrenal gland medulla (PC12) tumours are partially internalized by clathrin-mediated endocytosis, as demonstrated using pharmacological reduction (CPZ) and siRNA knockdown of clathrin [73]. In another report, the internalization of glioblastoma-derived exosomes involved non-classical, lipid raft-dependent endocytosis, and required ERK1/2-HSP27 signalling [75]. In this pathway, the negative regulation of ERK1/2 by caveolin-1 inhibited exosome endocytosis.

## 5. Exosome Intercellular Trafficking and Cellular Response

A wide range of stimulatory or inhibitory functional outcomes are shown to be induced following cellular interactions with exosomes, including proliferation, angiogenesis, apoptosis, cytokine production, immune system modulation, and invasion or metastasis. Whether or not a cellular response is elicited by the target cell likely depends on the mechanism of internalization.

Soluble, juxtacrine and fusion are most likely to end with a cellular response, as they do not directly engage the endosomal-lysosomal degradative pathway. Phagocytosis inevitably results in the fusion of the phagosome with lysosomes and the degradation of its contents.

The endocytosis of exosomes, whether by macropinocytosis, or receptor- or raft-mediated mechanisms, always results in the delivery of the vesicular cargo to the endosomal pathway. Early endosomes act as the sorting compartment. Those sent to late endosomes are then unidirectionally sent for degradation by fusion with lysosomes; meanwhile some proteins and fluids may be redirected back to the plasma membrane via recycling endosomes. However, even those sent to the late endosome may escape degradation by the trans-Golgi network.

It is suggested that the macropinocytotic clearance of exosomes by cells lacking an antigen-presenting capability may represent a mechanism for the degradation of the cellular membrane by immune cells in an immunologically ‘silent’ manner [72]. This implicates macropinocytosis as a mechanism of exosomal clearance rather than cell signalling. Thus, exactly which of these mechanisms represent bona fide mechanisms for exosome signalling and elicit a cellular response, and which are mechanisms of exosome clearance, needs to be delineated.

The internal transport network of phagosomes, macropinosomes, clathrin-coated vesicles and caveosomes requires a spectrum of cellular components. As an example, exosomes from chronic myeloid leukaemia cells were shown to require v-SNARE protein VAMP3/cellubrevin for the fusion of endosomes with autophagosomes [16], while VAMP7/TI-VAMP was required for fusion between the amphisome and lysosome. Similar dynamics of diffusion on the plasma membrane and internalization have been observed for natural and synthetic viruses [76, 77]. The internalization of exosomes from glioblastoma cells was dependent on ERK1/2 and HSP27 signalling [75]. Not surprisingly, intracellular filamentous actin [78] and microtubules [79] are also critical for the transport of exosome-containing vesicles within the cell following internalization.

Whether other cellular proteins known to be involved in endosomal transport—such as p38α, PKCδ, arrestins and syntaxins—are utilized when exosomes are internalized remains to be demonstrated.

## 6. Exosomes, T Cells and Therapy

Since exosomes are naturally present in the circulation of all individuals, and elicit effects that alter cellular functioning, they present a possible therapy in many disease settings. Molecular profiling and the functional analysis of tumour-derived exosomes show that they express death ligands and mediate apoptosis of CD8^+^ T cells (for a recent review, see [2]), while DC-derived exosomes promote CD4^+^ T cell proliferation and regulate T cell responses [80]. Placenta-derived exosomes regulate both functional characteristics, including mediating apoptosis, and inducing the proliferation and generation of regulatory T cells (Tregs) [48]. Yet the question remains as to what signals are required to elicit the desired therapeutic effect.

Manipulating the signals on the surface of exosomes or the mRNA/microRNA content of exosomes seems most plausible and has precedents. IL-10-treated exosomes from bone marrow-derived DCs prevented the onset of murine collagen-induced arthritis, and dampened the established disease [81], in a model shown to be associated with a robust and sustained T cell response to type II collagen [82]. Using this same model, exosomes derived from DCs genetically engineered to produce IL-4 suppressed the activity of T cells *in vivo,* via a MHC class II and FasL/Fas-dependent mechanism [83].

The reproducibility of using cell culture-derived exosomes for therapy remains an issue. The proteomic profiling of three independent batches of exosomes, from cultured human embryonic stem cell-derived mesenchymal stem cells (huES9.E1), identified ~400 unique proteins; however, only 154 proteins (~20%) were common to all three preparations [84]. Thus, the use of manipulated cultures to generate exosomes may result in a vast diversity of exosome protein and RNA content. With the relative success of lipid- and polymer-based nanomedicines, the extrapolation of the technology utilized for the extrusion, purification, analysis and labelling of these vesicles would most likely be required to generate therapeutic exosomes [21].

While this review updates what is currently known about the proteins involved in the interaction of (primarily) T cells with exosomes, and their internalization, further elucidation of these mechanisms is required, focusing on what governs the internalization mechanism used, and the signal transduction pathways leading to cellular responses. Once this is known, the therapeutic capability of exosomes is potentially limitless!

## 7. Compliance with Ethical Research Standards

The authors declare no conflicts of interest.
